# Methodological insights into multilevel analysis of individual heterogeneity and discriminatory accuracy: An empirical examination of the effects of strata configurations on between-stratum variance and of fixed effects across hierarchical levels

**DOI:** 10.1371/journal.pone.0297561

**Published:** 2024-03-18

**Authors:** Anne Laure Humbert

**Affiliations:** Centre for Diversity Policy Research and Practice, Oxford Brookes University, Oxford, United Kingdom; Chinese Academy of Medical Sciences and Peking Union Medical College, CHINA

## Abstract

This study aims to advance the Multilevel Analysis of Individual Heterogeneity and Discriminatory Accuracy (MAIHDA) approach by addressing two key questions. First, it investigates the impact of using increasingly complex combinations of variables to create intersectional strata on between-stratum variance, measured by the variance partitioning coefficients (VPCs). Second, it examines the stability of coefficients for fixed effects across models with an increasing number of hierarchical levels. The analysis is performed using data from a survey of over 42,000 respondents on the prevalence of gender-based violence in European research organisations conducted in 2022. Results indicate that the number of intersectional strata is not significantly related to the proportion of the total variance attributable to the variance between intersectional strata in the MAIHDA approach. Moreover, the coefficients remain relatively stable and consistent across models with increasing complexity, where levels about organisations and countries are added. The analysis concludes that the MAIHDA approach can be flexibly applied for different research purposes, either to better account for structures of power and inequality; or to provide intersectionality-sensitive estimates. The findings underscore the need for researchers to clarify the specific aims of using MAIHDA, whether descriptive or inferential, and highlight the approach’s versatility in addressing intersectionality within quantitative research. The study contributes to the literature by offering empirical evidence on the methodological considerations in applying the MAIHDA approach, thereby aiding in its more effective use for intersectional research.

## Introduction

Reconciling intersectionality with a quantitative approach is not without challenges. At the heart of the concept is the idea that different social identities intersect in unique ways to create structural inequalities and discrimination [1]. How power and privilege are experienced, via the lens of these myriads of intersecting identities, is regarded as incompatible with the somewhat crude categorisation that is invariably involved in any quantitative approach. The criticisms of quantitative methods in relation to intersectionality theory revolve around six main issues. First, it must be recognised that quantitative approaches tend to reflect dominant narratives, which shape the categories that are captured by data [2]. As a result, while more salient groups tend to be measured, this is at the expense of other minoritised groups that are not represented in data. Second, quantitative methods often default to assuming uniform experiences across a certain demographic group, the so-called ‘tyranny of the averages’ [3], that conflates ‘unity’ with group ‘uniformity’ [4]. This assumption overlooks the variance within these categories, posing the question of whether it is desirable to even consider that experiences should be regarded as universally identical within a particular group. Third, is the issue of the over-simplification of what are complex identities. Quantitative methods are inherently reductionist, often distilling complex realities into simpler, measurable variables [2, 5]. This leads to a decontextualisation of unique, individual identities and experiences. For instance, the interpretation of womanhood may vary substantially from one individual to another. In particularly, this asks the questions of whether it is possible to assume that being a woman will mean the same from one woman to another, even where they share the same other diversity traits. Fourth is the need to regard identities as being more than the sum of their parts. Identities can encapsulate additive traits, i.e. being a woman, being white. Identities can also encapsulate multiplicative traits, which is then concerned with being a white woman. However, as argued by Weldon [6], intersectionality is also about an additional ‘qualitative’ layer that goes beyond these additive or multiplicative aspects. What is certain is that identities rely of a process that is layered, raising questions about the capacity of quantitative methods to capture this complexity entirely. Fifth are concerns about statistical limitations. While certain quantitative methods attempt to incorporate interaction effects among different variables, there are inherent limitations related the number of interactions that can be effectively accounted for within a single model [7]. Sixth, this is exacerbated by the problem of achieving sufficient representation within intersectional groups. Where there are limited numbers, this can result in insufficient statistical power, thereby reducing the voice and visibility of these individuals in the data and, consequently, in the findings themselves [8].

**Fig 1:**
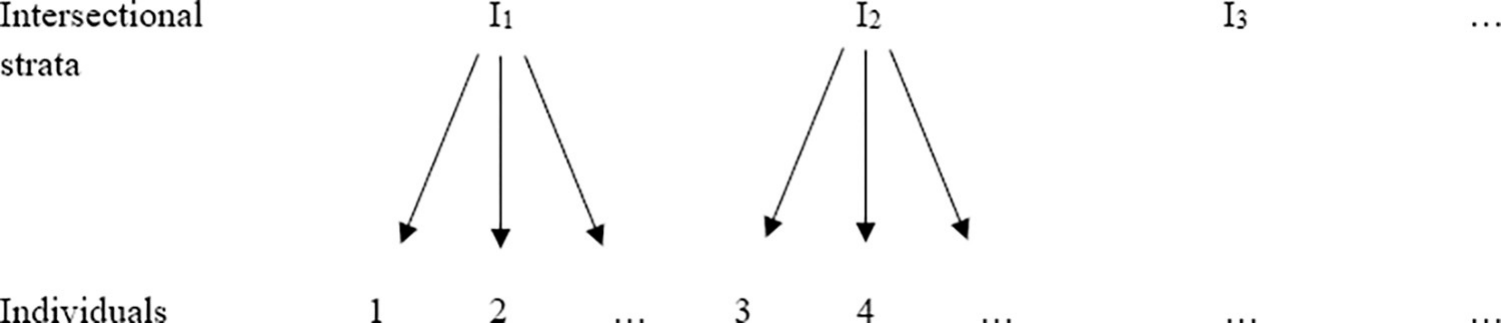
Unit diagram of a two-level structure.

These challenges underscore the need for more nuanced and flexible research strategies that can accurately portray the richness of intersectional identities and experiences. The latest advancements in quantitative intersectional research suggest integrating the interplay of various social determinants (such as age, gender identity, disability, etc.) into multi-level models using random effects, as opposed to the traditional method of including them as fixed effects in single-level regression models [7, 9–11]. Multi-level modelling approaches have been developed to deal with complex structures, including those where it is possible to identify *atomic units* nested into *higher level units* [12]. This new approach uses a multi-level model structure where intersectional strata combining all non-empty intersections of socio-demographic and functional diversity variables are placed at level 2 (Fig 1). The use of this complex structure is necessary to account for the non-independence of experiences of gender-based violence within different sets of social relations. In this article, we use the terminology ‘sets of social relations’, which Walby and colleagues [5] recommend instead of ‘categories’, to draw greater attention to power relations and inequalities between categories, and the actions of the powerful within categories. This terminology is thus more aligned to an understanding of intersectional inequalities that is structural, rather than merely linked to individual identity categories. Intersectional strata representing these sets of social relations are formed by creating membership groups along a predetermined set of characteristics. This intersectional multi-modelling approach, often referred to as multilevel analysis of individual heterogeneity and discriminatory accuracy (MAIHDA) [9] allows for an assessment of between-stratum variance; within-stratum heterogeneity; discriminatory accuracy by looking at the magnitude of within- and between-stratum variance relative to each other; how much between-stratum variance is explained by additive terms; and as with other modelling approaches, intersectional MAIHDA can also be used to estimate predicted probabilities or expected (mean) values of dependent variables for each stratum [8].

The use of intersectional multi-level modelling can be regarded as aligned with both the inter- and anti-categorical approaches to intersectionality outlined by McCall [2]. The creation of intersectional strata responds to the anti-categorical approach, which criticises the use of categorisation in the first place as it is seen to reify existing inequalities and fail to capture the complexity of different identities. Main effects provide information that is aligned with an inter-categorical approach by providing information on differences between groups on the basis of one or more individual membership criteria [13, 14]. In addition, the use of intersectional multi-level modelling is useful to shift the focus from inequalities located at the individual level, and towards inequalities stemming from an organisational/societal level imbalance of power at the structural level [5, 15].

What is not clear, however, is the number of variables that can be used for the construction of these intersectional strata. On the one hand, intersectionality theory would suggest, or even dictate, that it is desirable to create as many strata as possible, to attempt to reflect the complex reality of various intersecting identities. On the other hand, this presents a mathematical challenge in that where a stratum has too few observations, by construction, estimates of group parameters tend to the grand mean, providing little information about the group. This is due to the fact that higher level residuals are calculated as a mean raw residual (${\bar{r}}_{j}={\bar{y}}_{j}-{\hat{\beta }}_{0}$, i.e. the mean of $y_{ij}-{\hat{\beta }}_{0}$ for group *j*), and then multiplied by a shrinkage factor $k=\frac{{\hat{\sigma }}_{u}^{2}}{{\hat{\sigma }}_{u}^{2}+({\hat{\sigma }}_{e}^{2}/n_{j})}$ [16]. The shrinkage factor will adopt lower values where ${\hat{\sigma }}_{e}^{2}$ is large relative to ${\hat{\sigma }}_{u}^{2}$ (that is when variation is located more within individual heterogeneity than between strata) or where *n*_*j*_ (strata size) is small. Intersectional quantitative researchers using MAIHDA approaches agree that the method performs best with larger samples [10]. Yet, few guidelines are available on the desirable floor value to choose for *n*_*j*_, though Persmark and colleagues [13] imply adopting a minimum threshold of 50. Evans [8] for example also emphasises the need to ensure a minimum number of observations across most, if not all, stratum but does not specify any rule of thumbs. Milliren and colleagues [17] also suggest a lower bound of about 5 to 20, though they note that including some groupings with fewer observations is not problematic due to the shrinkage factor.

**Fig 2:**
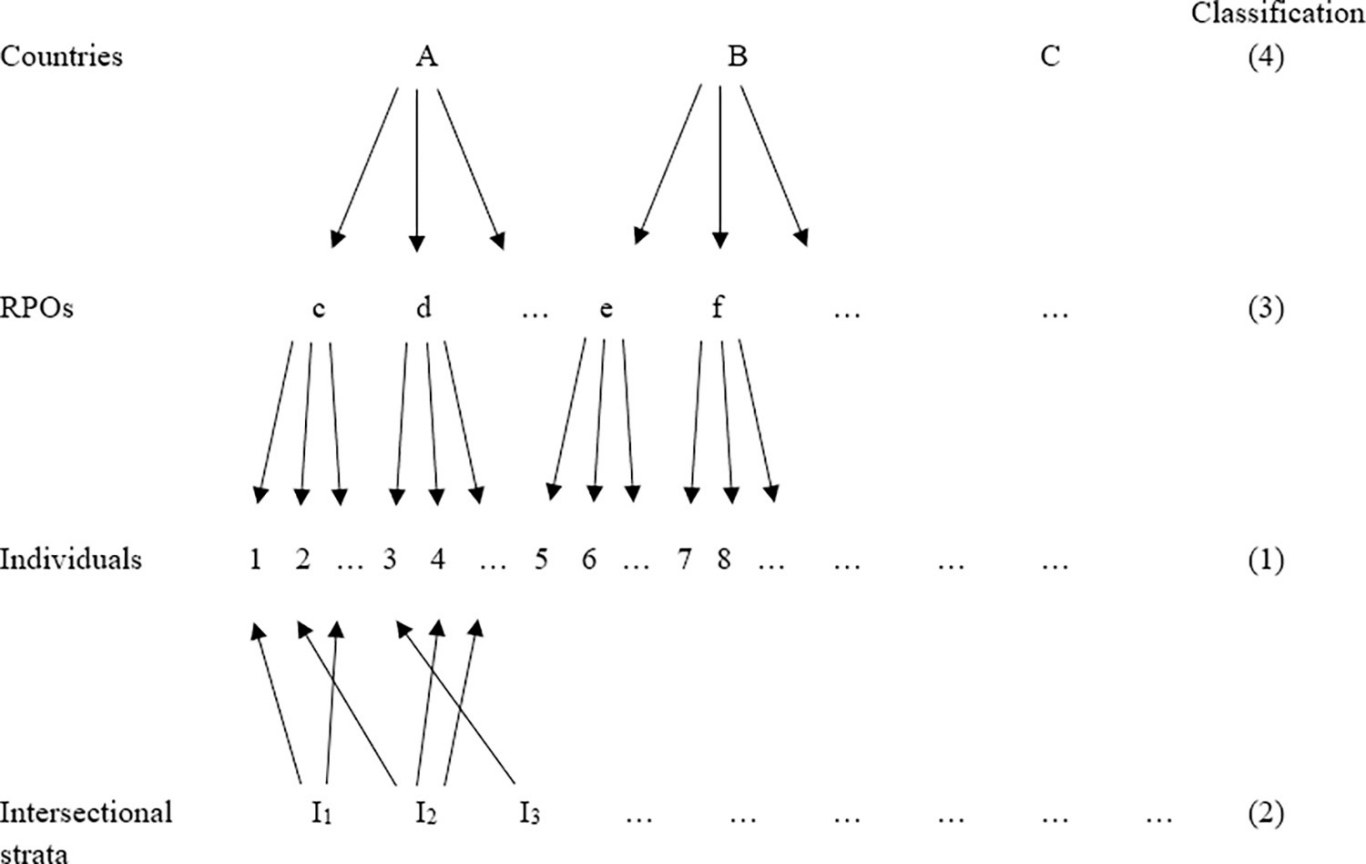
Unit diagram of a cross-classified structure.

In addition to these concerns about the number and size of intersectional strata, Persmark and colleagues [13] note as a limitation that they cannot take into account geographical location, which would a clear level to be used in modelling. Their concern is that it would necessarily reduce numbers within each stratum. However, this contextual information can be important, conceptually [15] as well as empirically [8]. To incorporate this additional level (or even more levels than one), it is necessary to consider that a nested structure is no longer sufficient to represent a complex reality, and that a cross-classified model is needed instead [17, 18]. This cross-classified structure (Fig 2) incorporates not only intersectional strata, but also organisational and national levels in which individuals are nested. The use of a cross-classified model, as opposed to a nested model, not only allows for the simultaneous analysis of multiple contexts but also mitigates the effects of potential ‘omitted context bias’ [17, 19]. While the recommendations for stratum size have been discussed in relation to nested model, these have seldom been examined in the context of cross-classified models. Exceptions are the work of Milliren and colleagues [17], though this work focuses on random effects rather than fixed effects, or Evans [20] which focuses on the robustness of the model after adding a contextual level.

The contribution of this paper is thus a methodological one by considering two questions in the application of the MAIHDA approach. The first aim is to test the extent to which increasingly complex combinations of variables used to compute intersectional strata, and thus increasing the number of intersectional strata while decreasing the number of observations within each stratum, has an effect on between-stratum variance measured by computing the variance partitioning coefficient (VPCs). The question of interest is whether between-strata variance is affected by the choice of different configurations of intersectional strata. The second aim is to examine whether coefficients, and thus information about the fixed effects of different traits used to construct intersectional strata, remain stable across models using an increasingly large number of levels, thus both increasing the number of strata and diminishing the number of observations within each stratum. The question of interest for this second aim is whether inferences about different sets of social relations are robust when not only considering intersectionality, but also contextual levels such as countries, and whether it is therefore feasible to also incorporate other classification levels (e.g. organisational, national) in a cross-classified structure given that this increases the number of strata, but decreases the size of each strata as each intersectional strata are then subdivided into groupings from other levels.

## Methods and sample

This paper contributes to literature on the MAIHDA approach by (1) illustrating whether adopting an increasing number of intersectional strata has an effect on between stratum and (2) whether coefficients change when using a cross-classification structure to also consider how individuals are nested both in institutions and countries. It relies on data on the prevalence of gender-based violence in research organisations, using a survey of n = 42,000+ respondents, which was designed to capture differences in the experiences of gender-based violence between different groups characterized by socio-demographic or functional diversity. The survey focused on experiences of gender-based violence in an academic context, and data were collected in 46 universities and other research organisations across 15 European countries between 17 January and 1 May 2022 [21]. The data collection was approved by the Leibniz Institute for the Social Sciences (GESIS) Ethics Committee (Approval number: 2021–7). Respondents to the survey provided written consent, and their answers were recorded anonymously with no possibility for reidentification. The data were accessed between 1 July and 15 November 2023 for the purpose of this analysis. As the dependent variable is dichotomous (having experienced any form of violence asked about or not), a logit link function is used.

The creation of intersectional strata is performed on the basis of eight variables: gender identity (women; men; non-binary or another gender identity not listed), trans status (current gender aligned with sex at birth; not aligned), sexual orientation (asexual; bisexual; heterosexual; homosexual; queer; another sexual orientation not listed), ethnic minoritised group (no; yes), disability or chronic illness (no; yes), age (up to 20 years; 21–25; 26–30; 31–35; 36–40; 41–45; 46–50; 51–55; 56–60; 60 and above); target group (staff; student); mobility (domestic; international). Intersectional strata are built iteratively, progressively building up to more combinations of these categories, and resulting in a total of n = 255 scenarios (the different combinations are listed in Table 1). When all variables are used in combination, this represents a total of 5,760 (3×2×6×2×2×10×2×2) distinct intersectional strata, though removing all empty strata yields n = 1,127 intersectional strata in the analysis. While there are on average about 34 individuals per intersectional strata in this scenario, it is important to note that this distribution is heavily skewed to the right. Only 61% of intersectional strata consist of more than a single individual, 22% have 10 or more individuals, 11% have 30 or more, and 8% have 50 or more. Using this high number of intersectional strata thus produces numbers in each stratum that are well below those used in the literature [see for example 7, 8].

**Table 1:** Number of strata, minimum, average, maximum sizes; Variance partitioning coefficients (VPCs) for the null models and additive main effects models by intersectional strata.

1: Gender identity 2: Trans status 3: Sexual orientation 4: Minority ethnic background 5: Disability or chronic illness 6: Age 7: Student or staff 8: Domestic or international	1	2	3	4	5	6	7	8	Number of strata	Minimum size (n of individuals)	Average size (n of individuals)	Maximum size (n of individuals)	VPC (null models)	VPC (additive main effects models)
1	x								3	1,120	13,994.00	28,149	8%	:
2		x							2	671	20,735.00	40,799	7%	:
3			x						6	537	6,700.30	32,475	2%	:
4				x					2	2,467	20,747.00	39,026	11%	:
5					x				2	4,462	20,581.00	36,700	20%	:
6						x			10	1,574	4,153.00	13,745	5%	:
7							x		2	17,907	21,015.00	21,122	20%	:
8								x	2	2,595	20,957.00	39,319	4%	:
1–2	x	x							6	76	6,909.70	27,971	5%	10%
1–3	x		x						18	78	2,232.70	21,853	3%	2%
1–4	x			x					6	173	6,910.80	26,378	11%	7%
1–5	x				x				6	310	6,855.30	24,642	8%	12%
1–6	x					x			30	26	1,383.70	9,521	8%	5%
1–7	x						x		6	287	6,997.20	16,562	10%	9%
1–8	x							x	6	109	6,979.50	26,482	5%	9%
2–3		x	x						12	60	3,314.80	32,320	3%	2%
2–4		x		x					4	81	10,252.80	38,075	2%	1%
2–5		x			x				4	213	10,174.30	35,933	13%	2%
2–6		x				x			20	4	2,050.70	13,224	6%	1%
2–7		x					x		4	99	10,367.50	23,193	9%	1%
2–8		x						x	4	51	10,342.80	38,209	3%	1%
3–4			x	x					12	52	3,318.70	30,522	2%	1%
3–5			x		x				12	124	3,292.30	29,013	3%	1%
3–6			x			x			60	3	663.80	9,641	5%	2%
3–7			x				x		12	107	3,350.20	17,094	5%	2%
3–8			x					x	12	43	3,342.30	30,503	1%	1%
4–5				x	x				4	422	10,188.50	34,384	21%	3%
4–6				x		x			20	57	2,051.80	12,783	4%	1%
4–7				x			x		4	801	10,373.30	22,153	8%	4%
4–8				x				x	4	520	10,348.30	36,940	2%	3%
5–6					x	x			20	195	2,035.50	12,257	7%	1%
5–7					x		x		4	2,008	10,291.00	21,186	12%	1%
5–8					x			x	4	240	10,265.00	34,329	5%	1%
6–7						x	x		20	18	2,076.60	13,326	11%	7%
6–8						x		x	20	54	2,071.80	13,017	4%	1%
7–8							x	x	4	1,033	10,479.00	22,533	6%	4%
1-2-3	x	x	x						36	1	1,104.80	21,805	3%	3%
1-2-4	x	x		x					12	7	3,416.80	26,234	3%	4%
1-2-5	x	x			x				12	20	3,390.60	24,525	6%	4%
1-2-6	x	x				x			60	1	683.50	9,451	7%	4%
1-2-7	x	x					x		12	18	3,454.80	16,433	9%	8%
1-2-8	x	x						x	12	5	3,446.60	26,321	4%	4%
1-3-4	x		x	x					36	10	1,105.90	20,626	3%	2%
1-3-5	x		x		x				36	16	1,097.10	19,467	4%	2%
1-3-6	x		x			x			174	1	228.80	6,654	6%	4%
1-3-7	x		x				x		36	13	1,116.30	11,737	5%	3%
1-3-8	x		x					x	36	3	1,113.80	20,643	3%	2%
1-4-5	x			x	x				12	74	3,394.30	23,204	4%	3%
1-4-6	x			x		x			60	4	683.70	8,917	6%	4%
1-4-7	x			x			x		12	44	3,455.40	15,367	7%	5%
1-4-8	x			x				x	12	38	3,447.40	25,069	4%	7%
1-5-6	x				x	x			60	6	678.20	8,516	8%	4%
1-5-7	x				x		x		12	69	3,427.70	14,637	10%	5%
1-5-8	x				x			x	12	34	3,419.30	23,161	5%	4%
1-6-7	x					x	x		60	3	691.90	9,259	15%	10%
1-6-8	x					x		x	60	3	690.30	9,018	7%	4%
1-7-8	x						x	x	12	37	3,489.80	15,512	7%	6%
2-3-4		x	x	x					24	6	1,642.60	30,403	2%	2%
2-3-5		x	x		x				24	13	1,630.50	28,900	4%	1%
2-3-6		x	x			x			107	1	368.30	9,597	5%	2%
2-3-7		x	x				x		24	4	1,657.40	17,002	4%	2%
2-3-8		x	x					x	24	4	1,653.60	30,361	2%	2%
2-4-5		x		x	x				8	34	5,039.80	33,731	4%	0%
2-4-6		x		x		x			40	1	1,014.40	12,349	4%	1%
2-4-7		x		x			x		8	16	5,126.40	21,405	5%	4%
2-4-8		x		x				x	8	18	5,114.80	36,044	2%	0%
2-5-6		x			x	x			39	1	1,032.50	11,914	7%	1%
2-5-7		x			x		x		8	29	5,087.10	20,601	10%	0%
2-5-8		x			x			x	8	18	5,075.10	33,621	5%	0%
2-6-7		x				x	x		39	1	1,051.60	12,821	13%	6%
2-6-8		x				x		x	36	1	1,136.70	12,528	5%	1%
2-7-8		x					x	x	8	11	5,171.40	21,683	5%	2%
3-4-5			x	x	x				24	14	1,633.00	27,393	3%	1%
3-4-6			x	x		x			119	1	331.60	9,044	5%	2%
3-4-7			x	x			x		24	11	1,659.30	15,876	4%	2%
3-4-8			x	x				x	24	14	1,655.60	28,942	2%	2%
3-5-6			x		x	x			120	1	326.20	8,882	6%	2%
3-5-7			x		x		x		24	25	1,646.10	15,488	6%	1%
3-5-8			x		x			x	24	11	1,642.30	27,219	3%	1%
3-6-7			x			x	x		117	1	340.40	9,340	12%	7%
3-6-8			x			x		x	115	1	345.60	9,172	5%	2%
3-7-8			x				x	x	24	15	1,671.20	16,016	4%	3%
4-5-6				x	x	x			40	21	1,008.00	11,473	5%	1%
4-5-7				x	x		x		8	156	5,094.30	19,619	6%	1%
4-5-8				x	x			x	8	80	5,082.10	32,500	3%	0%
4-6-7				x		x	x		40	3	1,025.90	12,391	10%	5%
4-6-8				x		x		x	40	6	1,023.60	12,210	4%	1%
4-7-8				x			x	x	8	164	5,174.10	20,982	4%	3%
5-6-7					x	x	x		40	4	1,017.80	11,872	13%	5%
5-6-8					x	x		x	40	7	1,015.40	11,584	5%	1%
5-7-8					x		x	x	8	99	5,132.50	19,782	6%	2%
6-7-8						x	x	x	40	2	1,035.90	12,628	10%	6%
1-2-3-4	x	x	x	x					67	1	588.30	20,583	3%	2%
1-2-3-5	x	x	x		x				70	1	559.00	19,432	5%	2%
1-2-3-6	x	x	x			x			256	1	153.90	6,638	6%	3%
1-2-3-7	x	x	x				x		69	1	576.40	11,705	5%	2%
1-2-3-8	x	x	x					x	67	1	592.30	20,602	3%	2%
1-2-4-5	x	x		x	x				24	2	1,679.60	23,105	4%	3%
1-2-4-6	x	x				x			105	1	386.40	8,860	6%	3%
1-2-4-7	x	x					x		24	1	1,708.40	15,260	6%	4%
1-2-4-8	x	x						x	24	1	1,704.50	24,934	3%	3%
1-2-5-6	x	x			x	x			110	1	366.00	8,470	9%	3%
1-2-5-7	x	x			x		x		24	4	1,695.30	14,551	10%	5%
1-2-5-8	x	x			x			x	24	1	1,691.30	23,057	5%	4%
1-2-6-7	x	x				x	x		105	1	390.60	9,189	14%	8%
1-2-6-8	x	x				x		x	104	1	393.40	8,954	7%	3%
1-2-7-8	x	x					x	x	23	2	1,798.20	15,398	7%	5%
1-3-4-5	x		x	x	x				72	2	544.20	18,434	3%	2%
1-3-4-6	x		x	x		x			308	1	128.10	6,257	6%	3%
1-3-4-7	x		x	x			x		72	1	552.90	10,953	5%	4%
1-3-4-8	x		x	x				x	72	1	551.70	19,658	3%	3%
1-3-5-6	x		x		x	x			327	1	119.70	6,117	7%	3%
1-3-5-7	x		x		x		x		72	2	548.50	10,618	6%	3%
1-3-5-8	x		x		x			x	72	1	547.30	18,369	4%	2%
1-3-6-7	x		x			x	x		302	1	131.80	6,462	12%	9%
1-3-6-8	x		x			x		x	292	1	136.10	6,335	6%	4%
1-3-7-8	x		x				x	x	72	1	556.90	11,025	6%	4%
1-4-5-6	x			x	x	x			118	1	341.60	8,009	7%	3%
1-4-5-7	x			x	x		x		24	13	1,697.10	13,636	7%	4%
1-4-5-8	x			x	x			x	24	12	1,693.20	22,022	4%	2%
1-4-6-7	x			x		x	x		118	1	347.60	8,668	13%	8%
1-4-6-8	x			x		x		x	118	1	346.90	8,508	7%	4%
1-4-7-8	x			x			x	x	24	11	1,723.70	14,564	6%	5%
1-5-6-7	x				x	x	x		117	1	347.80	8,275	15%	9%
1-5-6-8	x				x	x		x	118	1	344.10	8,046	8%	4%
1-5-7-8	x				x		x	x	24	7	1,709.70	13,692	7%	5%
1-6-7-8	x					x	x	x	115	1	360.10	8,774	13%	10%
2-3-4-5		x	x	x	x				48	1	808.90	27,302	3%	1%
2-3-4-6		x	x	x		x			186	1	210.00	9,006	5%	2%
2-3-4-7		x	x	x			x		48	1	821.30	15,807	5%	2%
2-3-4-8		x	x	x				x	48	1	819.50	28,828	2%	2%
2-3-5-6		x	x		x	x			193	1	200.90	8,846	6%	2%
2-3-5-7		x	x		x		x		48	2	815.20	15,418	6%	1%
2-3-5-8		x	x		x			x	48	1	813.40	27,115	4%	1%
2-3-6-7		x	x			x	x		181	1	217.70	9,297	11%	7%
2-3-6-8		x	x			x		x	178	1	220.90	9,131	5%	2%
2-3-7-8		x	x				x	x	47	1	844.40	15,935	4%	2%
2-4-5-6		x		x	x	x			73	1	546.60	11,175	6%	1%
2-4-5-7		x		x	x		x		16	7	2,519.90	19,114	5%	1%
2-4-5-8		x		x	x			x	16	8	2,514.10	31,883	3%	0%
2-4-6-7		x		x		x	x		72	1	563.60	11,968	10%	5%
2-4-6-8		x		x		x		x	69	1	586.80	11,795	4%	1%
2-4-7-8		x		x			x	x	16	4	2,557.40	20,272	4%	2%
2-5-6-7		x			x	x	x		70	1	575.20	11,540	13%	5%
2-5-6-8		x			x	x		x	69	1	582.20	11,264	6%	1%
2-5-7-8		x			x		x	x	16	1	2,537.60	19,238	6%	1%
2-6-7-8		x				x	x	x	68	1	601.80	12,152	11%	6%
3-4-5-6			x	x	x	x			224	1	173.40	8,361	5%	2%
3-4-5-7			x	x	x		x		48	3	816.50	14,449	5%	2%
3-4-5-8			x	x	x			x	48	3	814.70	25,943	3%	1%
3-4-6-7			x	x		x	x		208	1	189.70	8,762	10%	7%
3-4-6-8			x	x		x		x	214	1	184.00	8,661	5%	2%
3-4-7-8			x	x			x	x	48	4	827.80	15,049	5%	3%
3-5-6-7			x		x	x	x		218	1	179.60	8,601	11%	6%
3-5-6-8			x		x	x		x	214	1	182.50	8,430	6%	2%
3-5-7-8			x		x		x	x	48	1	821.20	14,494	5%	2%
3-6-7-8			x			x	x	x	208	1	191.10	8,886	11%	8%
4-5-6-7				x	x	x	x		79	1	510.40	11,108	11%	5%
4-5-6-8				x	x	x		x	80	1	502.90	10,936	5%	1%
4-5-7-8				x	x		x	x	16	27	2,541.10	18,550	5%	1%
4-6-7-8				x		x	x	x	79	1	518.30	11,842	9%	5%
5-6-7-8					x	x	x	x	78	1	520.70	11,228	11%	5%
1-2-3-4-5	x	x	x	x	x				124	1	313.10	18,402	4%	2%
1-2-3-4-6	x	x	x	x		x			400	1	97.70	6,242	6%	2%
1-2-3-4-7	x	x	x	x			x		122	1	323.10	10,925	6%	3%
1-2-3-4-8	x	x	x	x				x	117	1	336.20	19,619	3%	2%
1-2-3-5-6	x	x	x		x	x			434	1	89.30	6,105	8%	3%
1-2-3-5-7	x	x	x		x		x		128	1	305.70	10,594	7%	3%
1-2-3-5-8	x	x	x		x			x	122	1	320.00	18,340	4%	2%
1-2-3-6-7	x	x	x			x	x		390	1	101.00	6,446	12%	7%
1-2-3-6-8	x	x	x			x		x	382	1	102.90	6,321	6%	3%
1-2-3-7-8	x	x	x				x	x	121	1	328.00	11,000	5%	3%
1-2-4-5-6	x	x		x	x	x			183	1	218.00	7,967	8%	2%
1-2-4-5-7	x	x		x	x		x		47	1	857.70	13,561	7%	3%
1-2-4-5-8	x	x		x	x			x	45	1	893.70	21,930	4%	2%
1-2-4-6-7	x	x		x		x	x		175	1	231.80	8,611	12%	7%
1-2-4-6-8	x	x		x		x		x	175	1	231.30	8,456	6%	3%
1-2-4-7-8	x	x		x			x	x	45	1	909.10	14,466	6%	4%
1-2-5-6-7	x	x			x	x	x		180	1	223.70	8,229	14%	7%
1-2-5-6-8	x	x			x	x		x	181	1	222.00	8,005	8%	3%
1-2-5-7-8	x	x			x		x	x	45	1	902.00	13,617	7%	4%
1-2-6-7-8	x	x				x	x	x	170	1	240.70	8,710	12%	8%
1-3-4-5-6	x		x	x	x	x			526	1	73.80	5,765	7%	3%
1-3-4-5-7	x		x	x	x		x		142	1	275.90	9,936	6%	3%
1-3-4-5-8	x		x	x	x			x	138	1	283.30	17,550	4%	2%
1-3-4-6-7	x		x	x		x	x		484	1	81.50	6,077	11%	8%
1-3-4-6-8	x		x	x		x		x	483	1	81.50	5,993	6%	3%
1-3-4-7-8	x		x	x			x	x	140	1	283.70	10,399	6%	4%
1-3-5-6-7	x		x		x	x	x		513	1	76.30	5,939	12%	7%
1-3-5-6-8	x		x		x	x		x	501	1	77.90	5,809	7%	3%
1-3-5-7-8	x		x		x		x	x	136	1	289.70	9,963	7%	3%
1-3-6-7-8	x		x			x	x	x	469	1	84.70	6,150	11%	8%
1-4-5-6-7	x			x	x	x	x		218	1	184.90	7,778	13%	7%
1-4-5-6-8	x			x	x	x		x	216	1	186.20	7,623	7%	3%
1-4-5-7-8	x			x	x		x	x	48	2	846.60	12,902	6%	4%
1-4-6-7-8	x			x		x	x	x	215	1	190.40	8,274	12%	9%
1-5-6-7-8	x				x	x	x	x	207	1	196.10	7,823	14%	8%
2-3-4-5-6		x	x	x	x	x			320	1	120.20	8,329	6%	2%
2-3-4-5-7		x	x	x	x		x		90	1	431.40	14,394	5%	2%
2-3-4-5-8		x	x	x	x			x	90	1	430.40	25,855	3%	1%
2-3-4-6-7		x	x	x		x	x		288	1	135.60	8,724	11%	6%
2-3-4-6-8		x	x	x		x		x	295	1	132.10	8,626	5%	2%
2-3-4-7-8		x	x	x			x	x	89	1	442.00	14,985	5%	2%
2-3-5-6-7		x	x		x	x	x		307	1	126.30	8,566	11%	6%
2-3-5-6-8		x	x		x	x		x	303	1	127.70	8,396	6%	2%
2-3-5-7-8		x	x		x		x	x	87	1	448.80	14,431	6%	2%
2-3-6-7-8		x	x			x	x	x	289	1	136.10	8,846	11%	7%
2-4-5-6-7		x		x	x	x	x		127	1	314.20	10,819	12%	5%
2-4-5-6-8		x		x	x	x		x	124	1	321.10	10,652	5%	1%
2-4-5-7-8		x		x	x		x	x	31	1	1,297.60	18,070	5%	1%
2-4-6-7-8		x		x		x	x	x	124	1	326.50	11,437	9%	5%
2-5-6-7-8		x			x	x	x	x	119	1	337.60	10,917	11%	5%
3-4-5-6-7			x	x	x	x	x		370	1	105.00	8,095	10%	5%
3-4-5-6-8			x	x	x	x		x	360	1	107.70	7,990	6%	2%
3-4-5-7-8			x	x	x		x	x	95	1	411.60	13,677	6%	2%
3-4-6-7-8			x	x		x	x	x	349	1	112.80	8,392	10%	7%
3-5-6-7-8			x		x	x	x	x	353	1	110.70	8,164	11%	6%
4-5-6-7-8				x	x	x	x	x	149	1	270.00	10,595	10%	5%
1-2-3-4-5-6	x	x	x	x	x	x			623	1	61.80	5,753	7%	2%
1-2-3-4-5-7	x	x	x	x	x		x		211	1	184.00	9,915	6%	2%
1-2-3-4-5-8	x	x	x	x	x			x	199	1	194.70	17,521	4%	2%
1-2-3-4-6-7	x	x	x	x		x	x		570	1	68.50	6,062	11%	6%
1-2-3-4-6-8	x	x	x	x		x		x	572	1	68.10	5,980	6%	2%
1-2-3-4-7-8	x	x	x	x			x	x	197	1	199.70	10,375	6%	3%
1-2-3-5-6-7	x	x	x		x	x	x		618	1	62.70	5,927	12%	6%
1-2-3-5-6-8	x	x	x		x	x		x	609	1	63.50	5,799	7%	2%
1-2-3-5-7-8	x	x	x		x		x	x	206	1	189.50	9,945	7%	3%
1-2-3-6-7-8	x	x	x				x	x	555	1	70.90	6,136	11%	7%
1-2-4-5-6-7	x	x		x	x	x	x		288	1	138.50	7,736	13%	6%
1-2-4-5-6-8	x	x		x	x	x		x	289	1	137.80	7,586	7%	2%
1-2-4-5-7-8	x	x		x	x		x	x	82	1	490.50	12,834	6%	3%
1-2-4-6-7-8	x	x		x		x	x	x	275	1	147.20	8,222	11%	7%
1-2-5-6-7-8	x	x			x	x	x	x	280	1	143.50	7,782	13%	7%
1-3-4-5-6-7	x		x	x	x	x	x		763	1	50.90	5,596	11%	7%
1-3-4-5-6-8	x		x	x	x	x		x	750	1	51.70	5,510	7%	3%
1-3-4-5-7-8	x		x	x	x		x	x	250	1	156.40	9,422	7%	3%
1-3-4-6-7-8	x		x	x		x	x	x	710	1	55.40	5,818	11%	8%
1-3-5-6-7-8	x		x		x	x	x	x	738	1	52.90	5,638	11%	7%
1-4-5-6-7-8	x			x	x	x	x	x	363	1	110.80	7,407	13%	8%
2-3-4-5-6-7		x	x	x	x	x	x		471	1	81.70	8,063	10%	5%
2-3-4-5-6-8		x	x	x	x	x		x	466	1	82.40	7,960	6%	2%
2-3-4-5-7-8		x	x	x	x		x	x	156	1	248.30	13,625	5%	2%
2-3-4-6-7-8		x	x	x		x	x	x	439	1	88.80	8,357	10%	6%
2-3-5-6-7-8		x	x		x	x	x	x	455	1	85.00	8,131	10%	6%
2-4-5-6-7-8		x		x	x	x	x	x	208	1	191.40	10,319	10%	5%
3-4-5-6-7-8			x	x	x	x	x	x	551	1	70.30	7,737	10%	5%
1-2-3-4-5-6-7	x	x	x	x	x	x	x		851	1	45.20	5,584	11%	6%
1-2-3-4-5-6-8	x	x	x	x	x	x		x	839	1	45.80	5,500	7%	2%
1-2-3-4-5-7-8	x	x	x	x	x		x	x	319	1	121.40	9,404	7%	2%
1-2-3-4-6-7-8	x	x	x	x		x	x	x	786	1	49.60	5,805	10%	6%
1-2-3-5-6-7-8	x	x	x		x	x	x	x	837	1	46.20	5,628	11%	6%
1-2-4-5-6-7-8	x	x		x	x	x	x	x	433	1	91.90	7,370	12%	6%
1-3-4-5-6-7-8	x		x	x	x	x	x	x	1,042	1	37.20	5,346	11%	7%
2-3-4-5-6-7-8		x	x	x	x	x	x	x	663	1	57.90	7,707	10%	5%
1-2-3-4-5-6-7-8	x	x	x	x	x	x	x	x	1,120	1	34.30	5,336	11%	5%

The analytical approach consists of two parts. The first part examines the effects of increasingly complex intersectional strata on VPCs, using two related models. The first model is a null model (also known as the variance components model) which measures the extent to which differences in prevalence are located at the level of intersectional strata, i.e. how much difference there is *between* the strata. This is measured via the variance partitioning coefficient, calculated as $VPC=\frac{\sigma_{is}^{2}}{\sigma_{is}^{2}+3.29}$, where $\sigma_{is}^{2}$ is the variance between strata and where the variance between individuals within strata is estimated by π23≈3.29 which corresponds to the variance of the logistic distribution, under the assumption that prevalence can be regarded as a latent response. In the case of a two-level model, the variance partitioning coefficient corresponds to the intraclass correlation coefficient. The higher the VPC, the more variation is located between different groups. The second model is an additive main effects model (also known as random intercept model) which extends the first model by integrated the variables used to calculate intersectional strata as fixed effects for all models with at least two variables used in the intersectional strata (where there is only one variable, it is not desirable for it to be included both in the fixed and random part of the model), allowing VPC values to be computed. The first and second models are run iteratively, using increasing complex combinations for the intersectional strata. The results for each of the 255 combinations are provided in Table 1. The VPCs generated are then used in a simple OLS regression on the variables used to construct intersectional strata, and on intersectional strata characteristics (number, minimum size, average size, maximum size). Potential multicollinearity is checked by examining Variance Inflation Factors.

In the second part of the analysis, only the model with the greatest number of intersectional strata is retained, i.e. making use of the intersections provided by all the variables described above, and extended to a cross-classified model [18]. The complexity of the model is progressively increased, from a single-level logistic model (M1); to multi-level models including respectively a level for intersectional strata (M2), organisational level (M3) and national level (M4); and finally including all three levels in a cross-classified model (M5). These models are first estimated without any fixed effects (M1a to M5a), and subsequently with all the variables used to construct the intersectional strata as fixed effects (M1b to M5b). In the case of M1, the null model corresponds to the odds, while the additive main effects model consists of a logistic regression model. This analysis provides an examination of the stability of the estimates for the coefficients across increased complexity of the social structure considered in the model, and thus about whether their interpretation is similar.

All models are fitted through the external software package ‘runmlwin’ [22, 23] within Stata v17. The MCMC algorithm is used with a 5,000 iterations burn-in period followed by a monitoring period of 50,000 iterations and thinning every 50 iterations, with initial values provided by the IGLS (PQL2 method) parameter estimates [24].

## Results

The variance partitioning coefficients generated from the simulation of null models using increasingly granular intersectional strata have a mean of 7%, and range from 1% to 21%. As expected, the interclass correlation coefficients for the additive main effects models are lower overall, and range from 0% to 12%, with an average of 4%. The results for all possible combinations of intersectional strata are provided in Table 1. The results show that the variation in VPCs is related to the stratum specification used, with some of the characteristics used to construct the intersectional strata associated with higher VPCs at the intersectional level, as illustrated in Table 2.

**Table 2:** Regression of log-transformed variance partitioning coefficients on variables used to construct intersectional strata.

	VPCs (null models)			VPCs (additive main effects models)		
	Coefficient	SE	p-value	Coefficient	SE	p-value
Gender identity	0.1697600	0.0337298	<0.01	0.7651684	0.0590578	<0.01
Trans status	-0.0523960	0.0337298	0.12	-0.1827342	0.0590578	<0.01
Sexual orientation	-0.2161831	0.0337239	<0.01	-0.0151625	0.0589942	0.80
Ethnicity	-0.1417351	0.0337607	<0.01	-0.1425890	0.0590038	0.02
Disability/chronic illness	0.1771746	0.0337216	<0.01	-0.2806177	0.0589717	<0.01
Age	0.5319397	0.0337278	<0.01	0.5265723	0.0590254	<0.01
Student/staff	0.5740179	0.0337176	<0.01	0.7611514	0.0590006	<0.01
Domestic/international	-0.1430955	0.0337176	<0.01	-0.0305101	0.0590006	0.61
Constant	1.3903940	0.0512176	<0.01	0.3060449	0.0969203	<0.01
R^2^	0.731			0.653		
F-test	<0.01			<0.01		
Number of models	255			247		

**Fig 3:**
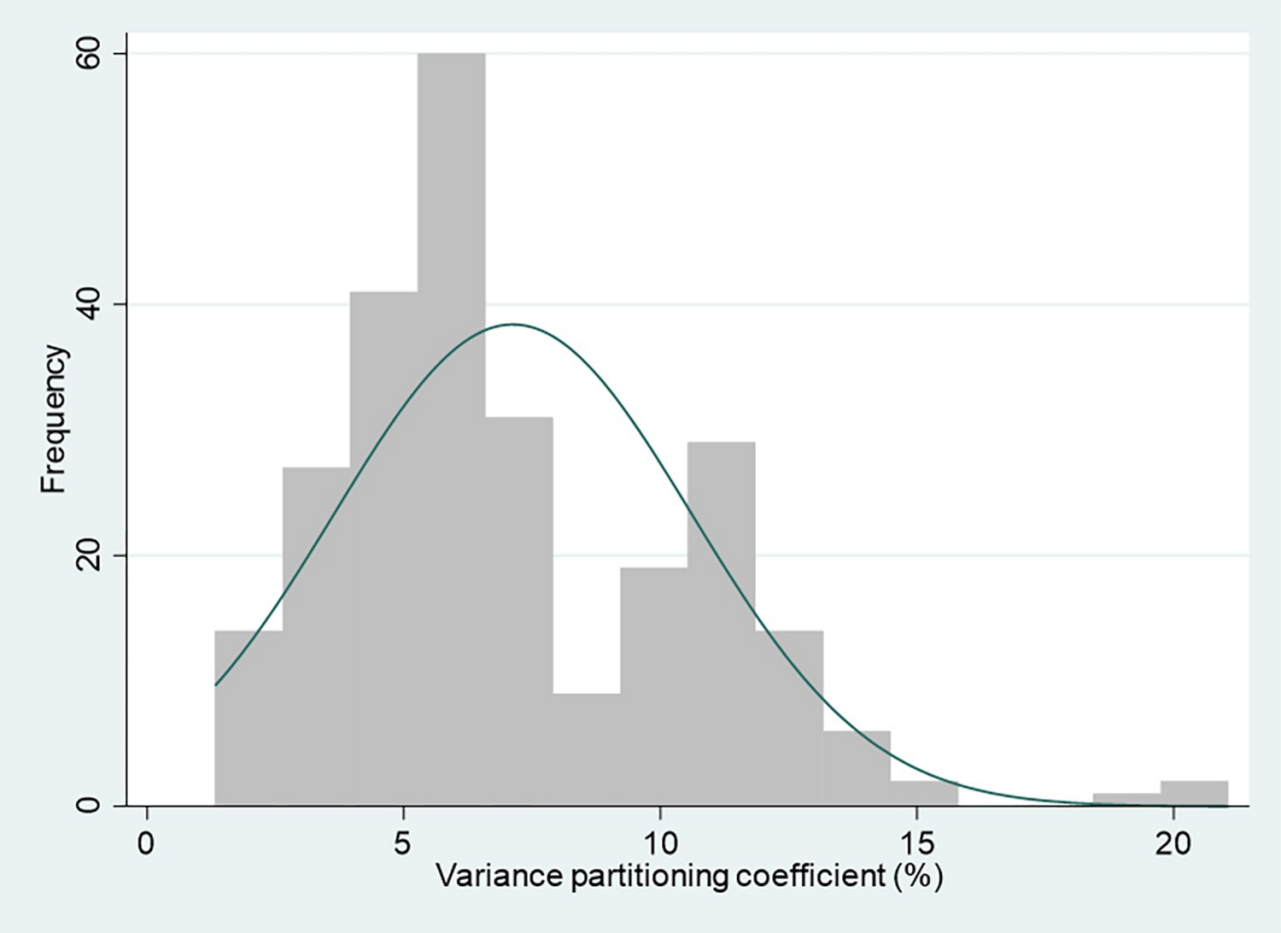
Variance partitioning coefficient distribution–null models.

**Fig 4:**
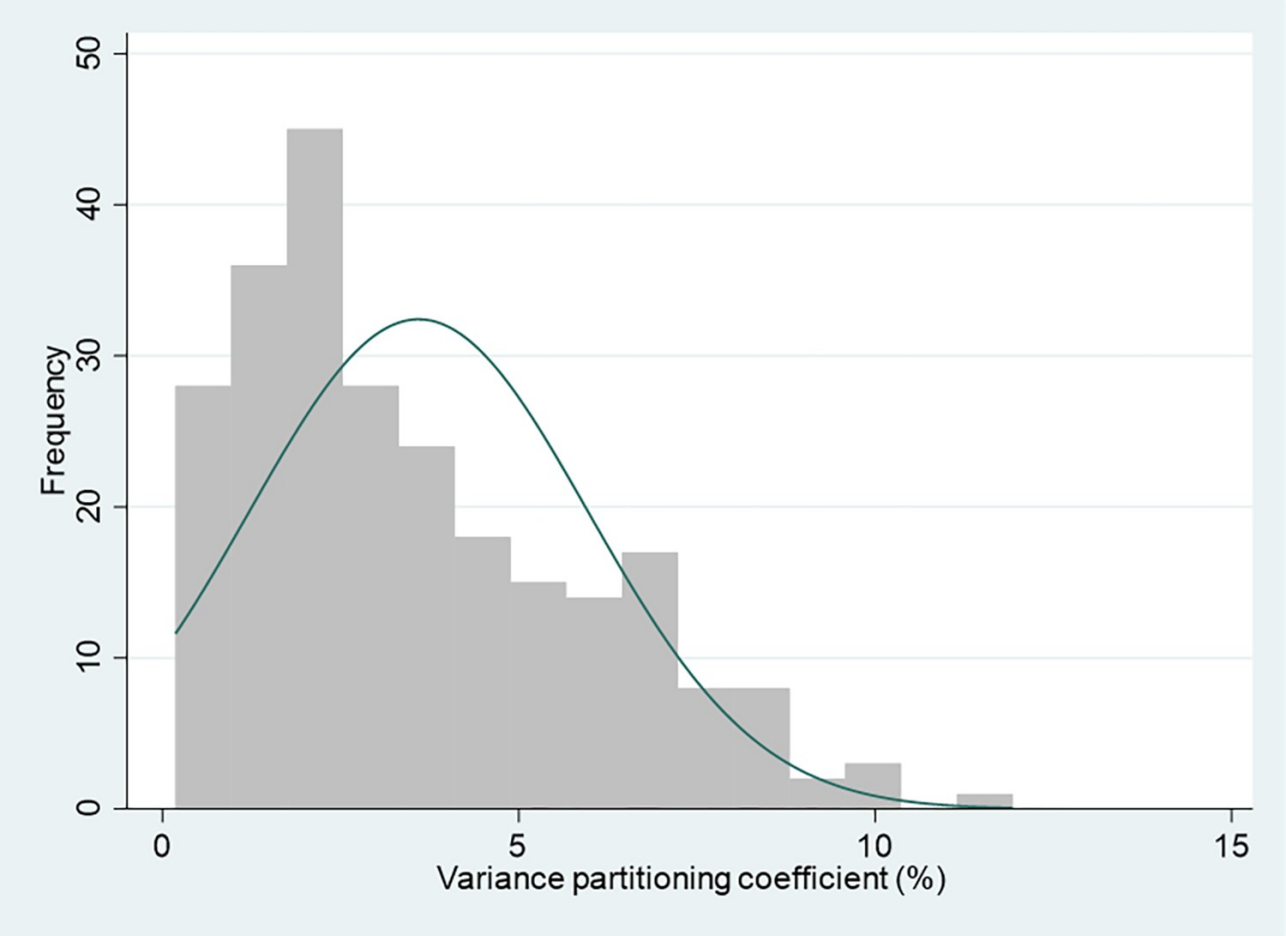
Variance partitioning coefficient distribution–additive main effects models.

To further explore how the variance partitioning coefficients relate the characteristics of these intersectional strata, simple regression models with log-transformed VPCs (expressed as percentages on a scale from 0 to 100) as the response variable are used. The VPCs were log-transformed due to a small skew in their distribution (Figs 3 and 4). Regression models were fitted using VPCs with and without this log transformation, with similar results. Only the log-transformed results are presented.

The models (Table 3) examine how different characteristics of intersectional strata relate to variance partitioning coefficients, to understand whether the size of intersectional strata affects VPCs. The results show that average size and maximum size are statistically significant. However, what is worth noting more than this statistical significance is the magnitude of these effects, as these are so small as to lose any practical significance. The number of intersectional strata is not related to the magnitude of variance partitioning coefficients.

**Table 3:** Regression of log-transformed variance partitioning coefficients on characteristics of intersectional strata.

	VPCs (null models)			VPCs (additive main effects models)		
	Coefficient	SE	p-value	Coefficient	SE	p-value
Number of strata	0.000095	0.000131	0.47	0.0003504	0.000258	0.18
Minimum size	-0.000026	0.000023	0.26	-0.0004774	0.000357	0.18
Average size	0.000107	0.000011	<0.01	0.0001082	0.000034	<0.01
Maximum size	-0.000063	0.000004	<0.01	-0.0000604	0.000009	<0.01
Constant	2.553498	0.071218	<0.01	1.6934450	0.141450	<0.01
R^2^	0.555			0.258		
F-test	<0.01			<0.01		
Number of models	255			247		

The aim of the second part of the analysis is to understand whether the use of an increasing number of levels in a cross-classified structure affects conclusions that could be reached, owing to increasingly small strata when intersectional strata are broken down further by country and institution (Tables 4 and 5). All models suggest that there is very little variation in the level of gender-based violence at country or institutional level. Some variation is present across intersectional strata, when covariates are not included. However, once fixed effects are added, there remains very little variation at the intersectional of the sets of social relations included in the analysis.

**Table 4:** Null models for a single-level, two-level (x3), and cross-classified three-level.

	M1a		M2a		M3a		M4a		M5a	
Odds ratio	1.616	n/a	2.153	<0.01	1.873	<0.01	1.777	<0.01	1.984	<0.01
$\sigma_{v}^{2}$							0.135		0.057	
$\sigma_{u}^{2}$					0.124				0.066	
$\sigma_{is}^{2}$			0.414						0.319	
*VPCv*							0.040		0.015	
*VPCu*					0.036				0.018	
*VPCis*			0.111						0.085	
Number of countries (*v*)							15		15	
Number of RPOs (*u*)					46				46	
Number of intersectional strata (*is*)			1,120						6,263	
Number of individuals	42,029		38,396		38,396		38,396		38,396	

**Table 5:** Additive main effects models for a single-level, two-level (x3), and cross-classified three-level.

	M1b		M2b		M3b		M4b		M5b	
Constant	2.504	<0.01	2.128	<0.01	2.124	<0.01	2.291	<0.01	2.096	<0.01
Student	0.593	<0.01	0.642	<0.01	0.697	<0.01	0.664	<0.01	0.696	<0.01
Woman	1.709	<0.01	1.622	<0.01	1.732	<0.01	1.726	<0.01	1.725	<0.01
Non-binary	1.788	<0.01	1.782	<0.01	1.860	<0.01	1.857	<0.01	1.850	<0.01
Trans	1.196	0.16	1.202	0.10	1.193	0.09	1.209	0.09	1.190	0.10
Disabled	1.545	<0.01	1.622	<0.01	1.559	<0.01	1.561	<0.01	1.603	<0.01
Minority ethnic	1.400	<0.01	1.436	<0.01	1.362	<0.01	1.362	<0.01	1.373	<0.01
Asexual	0.955	0.60	1.039	0.38	0.970	0.35	0.980	0.39	0.974	0.37
Bisexual	1.473	<0.01	1.453	<0.01	1.485	<0.01	1.489	<0.01	1.493	<0.01
Homosexual	1.338	<0.01	1.435	<0.01	1.327	<0.01	1.321	<0.01	1.352	<0.01
Queer	1.542	<0.01	1.736	<0.01	1.561	<0.01	1.563	<0.01	1.621	<0.01
Another sexual orientation	1.223	0.06	1.350	<0.01	1.218	0.03	1.223	0.03	1.257	0.03
International	1.027	0.57	1.039	0.29	0.946	0.11	0.947	0.13	0.967	0.26
Age (mc)	0.980	<0.01	0.983	<0.01	0.983	<0.01	0.984	<0.01	0.982	<0.01
Time at RPO (mc)	1.084	<0.01	1.088	<0.01	1.079	<0.01	1.079	<0.01	1.082	<0.01
$\sigma_{v}^{2}$							0.076		0.043	
$\sigma_{u}^{2}$					0.069				0.031	
$\sigma_{is}^{2}$			0.065						0.099	
*VPCv*							0.022		0.012	
*VPCu*					0.020				0.009	
*VPCis*			0.019						0.029	
Number of countries (*v*)							15		15	
Number of RPOs (*u*)					46				46	
Number of intersectional strata (*is*)			1,105						6,205	
Number of individuals	38,095		38,095		38,095		38,095		38,095	

The analysis examines whether the use of increasingly complex strata, here not only by intersectional sets but also countries and institutions, affects the results. This might be the case as doing so means using more numerous groups, hence relying on strata with small membership. In M5b, for example, the model incorporates 6,205 strata, which range from size 1 to 923, and with an average of just 6.1. An examination of the coefficients across M1b to M5b demonstrates that interpretations about the odds of experiencing gender-based violence for different group characteristics (considering all other variables, strata membership, and noting possible omitted variables bias) remain relatively stable. It can be concluded that for the purpose of inferences about a population, the use of very granular strata is thus appropriate. Going even further, it is possible to argue that it is not only appropriate but in fact desirable, because this approach is most aligned to the principles of analysing data both intersectionally and in context [15].

## Conclusion

The paper contributes to scholarship on the Multilevel Analysis of Individual Heterogeneity and Discriminatory Accuracy (MAIHDA) approach by exploring two main questions. The first question examines how using increasingly complex combinations of variables to create intersectional strata affects between-stratum variance, as measured by the variance partitioning coefficient (VPCs). The results show that the number of intersectional strata used appears to be unrelated to between stratum variance. The second question investigates the stability of coefficients for the fixed effects of different characteristics used in constructing intersectional strata across models in a cross-classified model with additional levels. This aims to assess the robustness of inferences about social relations when considering not just intersectionality but also other contextual levels like organisations and countries. The results suggest that increasing the number of levels, and as a result increasing the number of strata and diminishing the number of individuals therein, does not hinder the potential conclusions that can be reached about the effects of individual strand of identities. Further work should consider whether the results obtained in this analysis can be replicated using a simulated dataset, as this would provide insights from knowing with certainty about underlying relationships and how the results would appear in different parameter spaces. In addition, these results were obtained on the basis of a binary outcome, and ought to be replicated for other types of response variables including continuous normally distributed outcomes as well as ordinal or non-normally distributed outcomes.

What can be concluded more generally from these results? These results do not mean that the advice given in the literature [8, 13] to pay attention to the structure of intersectional strata should not be heeded. Instead, it points to the importance of stressing the ‘*and*’ in the MAIHDA approach: Multilevel Analysis of Individual Heterogeneity *and* Discriminatory Accuracy. There are different purposes for which the approach can be used, and which should therefore be clarified explicitly at the outset of any analysis. The results presented here show that if the purpose is to include interaction terms to better account for structures of power and inequalities, then a greater number of intersectional strata is possible, and indeed desirable in line with the transformational aim intersectionality theory [4, 5]. Using fewer intersectional strata is a loss of information that, from an intersectional perspective, is hard to justify as the aim should be to provide data about all categories, and particularly the most minoritised ones, which also tend to be the smallest strata. However, if the aim is to provide intersectionality-sensitive estimates, for example here of the prevalence of gender-based violence across different sets of social relations, then as suggested in the literature, a large number of intersectional strata may not be informative because of the shrinkage property, whereby small intersectional strata’s estimates revert to the grand mean. As Bell [25] emphasises, the two main aims of MAIHDA–that is either to understand which grounds and intersectional matter, and provide specific measures for these intersections–have yet to be fully worked out in relation to their implications for policy and practice. This analysis reminds us of the need to clarify and remember the purpose for which MAIHDA is used, and whether the aim is descriptive (classification to understand *if* intersectionality matters) or inferential (*how* to address inequalities through understand the axes along which they operate).
